# Probiotic-fortified functional foods: integrating nutrient delivery and gut health benefits

**DOI:** 10.3389/fnut.2026.1815558

**Published:** 2026-04-20

**Authors:** Saleh A. Alsanie

**Affiliations:** 1 Department of Basic Health Sciences, College of Applied Medical Sciences, Qassim University, Al-Melida, Saudi Arabia; 2 Department of Clinical Nutrition, Medical City, Qassim University, Al-Melida, Saudi Arabia

**Keywords:** fortification, functional foods, gut health, health benefits, microbiota, nutrient delivery, probiotics

## Abstract

Foods fortified with probiotics are a fast-emerging field at the crossroads of food technology, nutritional biochemistry and microbiome science. The increased interest in the gut microbiota as a key controller of host metabolism, immunity and overall homeostasis has led to the creation of diets that provide key nutrients with live and beneficial microbes. Compared to the conventional dietary supplementation, there are improved microbe stability, bioavailability, and microbe-nutrient interactions of probiotic fortification of food matrices. This review is a summary of the literature on the impact of probiotics on the host immunological and metabolic signalling pathways, intestinal barrier functioning, and gut microbiota composition. The biological mechanisms of interaction of probiotics with the intestinal microenvironment are specifically focused on the production of short-chain fatty acids, expulsion of pathogens, the regulation of immune cells, and the communication of the gut-brain axis. New information that can be used to correlate the administration of probiotics with the improvement of gastrointestinal health, systemic inflammation, metabolic maintenance and neurobehavioral phenotypes is narratively synthesized based on available preclinical and clinical evidence. The opportunities of probiotic-enriched functional foods have been highlighted in this review as a strategic tool of disease prevention and health promotion in the context of the mechanistic knowledge in combination with translational health outcomes. The complexity in the interactions between microbial delivery systems and host physiology is the clue to the best efficacy, safety and the future innovation in the development of functional foods.

## Graphical abstract

**Figure:**
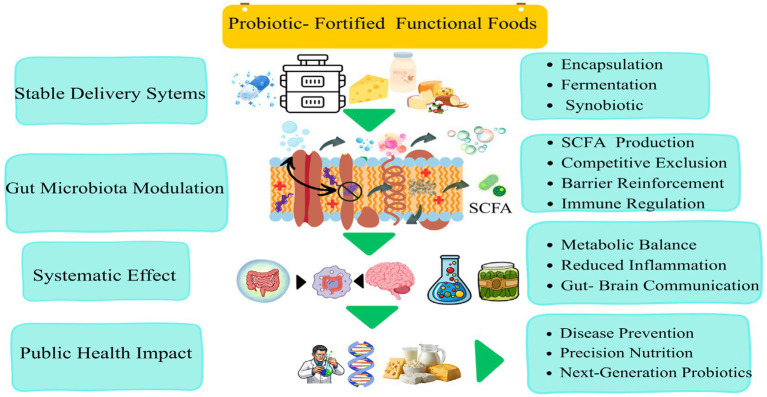

## 1 Introduction

Human gastrointestinal tract contains a very complex and dynamic microbial ecosystem that is essential in the metabolism of nutrients, immune development, maintenance of epithelial barriers and systemic physiological regulation. Recent developments in metagenomics, metabolomics, and systems biology have shown that microbial composition disruptions, also known as dysbiosis, are related to a broad spectrum of chronic diseases, such as inflammatory bowel disease, obesity, type 2 diabetes, metabolic syndrome, and neuropsychiatric diseases (1–3).

Probiotics can be described as live microorganisms that, when given in sufficient doses, produce a health effect on the host (4, 5). Importantly, probiotic effects are highly strain-specific and dose-dependent, and therefore cannot be generalized across species or genera (6, 7). Clinical outcomes vary significantly depending on the administered strain, its viability at the time of consumption, and the dosage (typically expressed as colony-forming units, CFU) (8). For instance, *Lactobacillus rhamnosus* GG has demonstrated efficacy in preventing antibiotic-associated diarrhea (9), whereas *Bifidobacterium longum* 1714 has been associated with psychobiotic effects in stress modulation (10). Moreover, therapeutic thresholds often range between 10^8^ and 10^11^ CFU/day, depending on the clinical indication and host condition (11). This highlights the necessity of precise strain identification and dose optimization in functional food design and clinical translation. Widely used genera in food include Lactobacillus (recently reclassified in many genera), Bifidobacterium, *Saccharomyces*, and strains of *Bacillus* and *Escherichia coli*. Despite the widespread availability of probiotic supplements, there has been an increasing interest in incorporating probiotics into functional food matrices to enhance delivery effectiveness, increase consumer compliance, and enable probiotics to work in concert with other dietary components (12).

Probiotic-enriched functional foods are not just a nutrition plan, but aim to achieve a particular physiological effect. These products can include bioactive peptides, vitamins, minerals, polyphenols, or probiotics with prebiotics as integrated delivery systems that work together to control human reactions and microbial metabolism (13, 14). These interventions can be successful depending on a variety of variables, such as strain specificity, resistance to processing and storage, resistance to gastric acidity, and the ability to colonize or temporarily interact with the host gut environment.

Recent studies have moved from the descriptive correlations to the mechanistic knowledge explaining how probiotic strains mediate host–microbe interactions on a molecular, and cellular scale (12, 15). These processes involve competitive exclusion of pathogens, improvement of the mucosal barrier activity, regulation of innate and adaptive immunity pathways, and regulated production of microbial metabolites (16, 17). Besides, there is growing clinical evidence that probiotic-enriched foods might add to prevention measures against diseases, as well as to long-term health optimization (2, 14).

This review provides a comprehensive analysis of the health consequences and mechanisms of action of probiotics, which will serve as the conceptual foundation for future discussions of nutrition delivery methods and technical developments in probiotic fortification.

### 1.1 Literature search strategy

A thorough literature search was carried out for this narrative review utilising databases such as PubMed, Scopus, and Web of Science up to 2026. Keywords included ‘probiotics’, ‘functional foods’, ‘gut microbiota’, ‘encapsulation’, and ‘probiotic delivery systems.’ Priority was given to recent systematic reviews, meta-analyses, and randomized controlled trials, supplemented by mechanistic *in vitro* and animal studies where relevant. This review follows a narrative synthesis approach rather than a formal systematic review; therefore, PRISMA guidelines were not strictly applied.

## 2 Probiotics: mechanisms of action and health benefits

**Figure 1:**
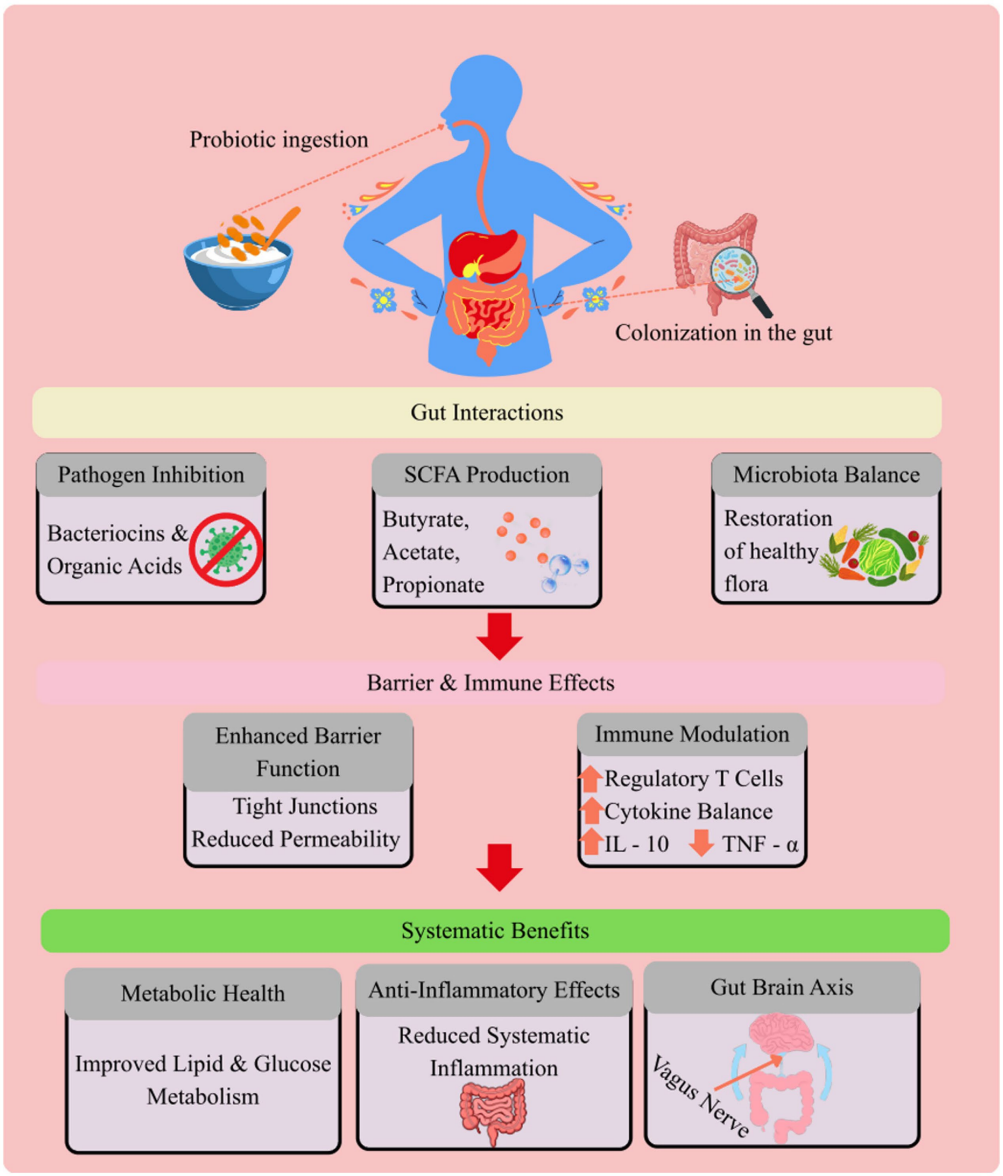
Mechanisms of probiotic action in gut health, including microbial competition, short-chain fatty acid production, immune modulation, and enhancement of intestinal barrier integrity, and their interactions with host physiological pathways.

The positive effects of probiotics are associated with intricate interactions of microorganisms introduced, microbiota present in the gut, intestine epithelial cells, and immune components (Figure 1). Such interactions can be divided into four main mechanistic realms, including microbial competition and ecological modulation, metabolic activity and metabolite production, immune regulation, and enhancement of barrier functions.

Recent multi-omics studies have also delineated further that probiotic effects cannot be limited to taxonomic alterations but also to functional metabolic restructuring of the gut ecosystem. Metagenomics and shotgun metabolomics studies show that the administration of probiotics can modify carbs, bile acid conversion, and inflammatory signalling pathway gene expression patterns despite seemingly minor changes in composition (12, 18, 19). These findings support the unique notion that a more accurate measure of probiotic efficacy is functional metabolic output rather than microbial abundance alone. Epithelial defence mechanisms are further supported by transcriptome profiling of intestinal epithelial cells, which reveals a regulation of NF-kB signalling, mucin gene expression (MUC2), and antimicrobial peptide synthesis (17, 20).

### 2.1 Microbial modulation and colonization resistance

Competitive exclusion, nutrient competition, and antimicrobial compound secretion (e.g., bacteriocins, hydrogen peroxide, organic acids, etc.) are all ways in which probiotics affect gut microbial ecology (21, 22). Probiotic strains prevent opportunistic pathogen (Such as *Clostridioides difficile* and pathogenic *Escherichia coli*) colonization by decreasing luminal pH by producing lactic acid and preventing the adhesion of pathogens to epithelial receptors.

Moreover, probiotics could facilitate positive cross-feeding. As an example, lactic acid bacteria are capable of producing lactate that can be converted to butyrate by commensal anaerobes, thus improving colonic health (23); however, this cross-feeding interaction is highly strain and community-dependent, varying according to microbial composition and substrate availability within the gut ecosystem (24) (Figure 1).

### 2.2 Gut health: microbiota balance and barrier integrity

Regaining and sustaining gut microbial balance is considered to be one of the most regularly documented advantages of probiotics. Dysbiosis, which is a diminished diversity of microbes and excess of pathobionts, has been linked with inflammatory and metabolic diseases. It has been demonstrated that probiotics can raise the relative abundance of beneficial taxa, including *Bifidobacterium* and *Faecalibacterium prausnitzii*, and decrease inflammatory microbial symptoms (Figure 1) (25).

In addition to modulations at the compositional level, probiotics improve the intestinal barrier integrity. The gut epithelial layer is a selective barrier, which is regulated by tight junction protein such as claudins, occludin and zonula occludens-1. Some probiotic strains enhance the expression of tight junction genes and lower intestinal permeability to inhibit the translocation of endotoxins like lipopolysaccharide (Figure 1) (16, 26).

Additionally, colonocytes take up energy from probiotic-derived short-chain fatty acids (SCFAs), particularly butyrate (Figure 1), which also affect anti-inflammatory signal transduction by inhibiting histone deacetylase and activating G-protein-coupled receptors. The combination of these mechanisms reinforces the mucosal homeostasis and prevents inflammatory damage (27).

Besides SCFA-mediated histone deacetylase inhibition, recent studies also emphasize the contribution of probiotics to the control of the aryl hydrocarbon receptor (AhR) signaling, one of the pathways that play an essential role in intestinal immune homeostasis and epithelial renewal (28, 29). Some of the Lactobacillus strains generate indole derivatives of tryptophan metabolism that stimulates AhR, leading to IL-22 release and repairing the mucosa. Moreover, probiotics have been found to regulate the expression of zonulin, which regulates the tight junction permeability, and which has been implicated in metabolic and autoimmune diseases (30). These new avenues expand the knowledge of the role that probiotics play in the strengthening of barriers in addition to the classical tight-junction protein up-regulation.

### 2.3 Systemic effects: immunity, metabolism, and the gut-brain axis

Though the probiotic actions begin in the gut lumen, their effects spread systemically via immune, metabolic and neuroendocrine pathways.

#### 2.3.1 Immune modulation

Pattern recognition receptors (Toll-like receptors) on epithelial and immune cells interact with probiotics and regulate the production of cytokines and differentiation of T-cells. Some of those strains stimulate the growth of regulatory T cells and the increase of anti-inflammatory cytokines (e.g., IL-10) and the decrease of pro-inflammatory mediators (e.g., TNF-*α*, IL-6) (Figure 1) (31, 32). These immunomodulatory functions form the basis of their potential therapeutic use in allergic diseases and inflammatory bowel diseases.

#### 2.3.2 Metabolic regulation

The emerging evidence indicates that probiotics have the ability to modulate glucose homeostasis, lipid metabolism, and adiposity. The suggested mechanisms are the ability to regulate the bile acid metabolism, enhance insulin sensitivity via SCFA-mediated signalling, and decelerate endotoxemia-related inflammation (Figure 1) (33, 34).

#### 2.3.3 Gut-brain axis

The gut microbiota has a two-way communication with the central nervous system via neural (vagus nerve) (Figure 1), endocrine, and immune pathways. Probiotic strains that can be called “psychobiotics” have been shown to have potential in anxiety and depressive symptom reduction, potentially through regulation of neurotransmitter precursors, tryptophan metabolism, and inflammatory signalling (1, 35). Although evidence is still developing, these results suggest the systemic scope of gut-targeted interventions. In addition to modulating neurotransmitter precursors, psychobiotic strains are linked to changes in hypothalamic–pituitary–adrenal (HPA) axis functions. Randomized controlled trials will show decreased cortisol outputs and enhanced stress-resistance after the administration of multi-strain probiotic supplement (10, 36). Mechanistically, SCFAs could also modulate the maturation of microglia and neuroinflammatory processes, which could be the biological explanation of positive effects on anxiety and depressive symptoms (1, 37). Moreover, the role of vagal nerve stimulation seems to be central to the behavioral effects of probiotics because vagotomy nullifies anxiolytic behavior in preclinical paradigms (38). Nevertheless, current evidence remains preliminary and requires validation in large-scale human trials.

### 2.4 Disease prevention and therapeutic potential

Probiotics have been explored as preventive or adjunct treatment in various context of disease.

#### 2.4.1 Gastrointestinal disorders

There is strong evidence that probiotics prevent antibiotic-associated diarrhea and decreases recurrence of *Clostridioides difficile* infection (39). In inflammatory bowel illness, particularly ulcerative colitis, several strains have demonstrated a modest level of efficacy in preserving remission.

Recent umbrella reviews and meta-analyses also affirm that the preventive effect against antibiotic-associated diarrhoea is strain-specific with *Lactobacillus rhamnosus* GG and *Saccharomyces boulardii* showing the most consistent clinical effects (40). The probiotics decrease pathogen overgrowth through a mechanistic action of restoring microbial diversity, generating antimicrobial compounds and increasing mucosal immune responses. Probiotics have the potential to prevent toxin-mediated epithelial injury and decrease recurrence in the context of *Clostridioides difficile* infection by stabilizing the colonization resistance process.

Some multi-strain probiotic preparations like VSL#3 have shown evidence of improvement in clinical remission and mucosal healing in inflammatory bowel disease (IBD), especially ulcerative colitis as adjunctive treatment. VSL#3 is a multi-strain probiotic known for its positive impact on gut health. However, it is important to note that the formulation of VSL#3 changed after 2016. The original De Simone Formulation, which was marketed under the VSL#3^®^ trademark, was available only until 2016 (41). The suggested mechanisms are the down-regulation of NF-kB-mediated inflammatory pathways, the control of dendritic cell activity, and the enhanced production of anti-inflammatory cytokines, including IL-10 (5, 17). Nevertheless, the therapeutic reactions are still diverse, and the theme of selection of strains individually and disease-specific targeting is significant.

#### 2.4.2 Irritable bowel syndrome

Meta-analyses have indicated a positive change in abdominal pain, bloating, and global symptom scores with strain-specific probiotic preparations, but there is still heterogeneity in the outcomes.

Recent randomized control trials indicate that probiotics can have an effect on visceral hypersensitivity, intestinal motility, and low-grade mucosal inflammation- critical pathophysiological characteristics of irritable bowel syndrome (IBS). Some *Bifidobacterium* and *Lactobacillus* strains have been shown to alter the gut-brain signalling pathways, which may alleviate the severity of symptoms by acting on serotonin metabolism and vagal nerve signalling (1, 35).

Moreover, probiotics have the potential to decrease intestinal permeability and correct the observed changes in microbial fermentation patterns in patients with IBS and, as a result, reduce the level of gas production and luminal distension. Although there are encouraging results, the inconsistent clinical results are due to variability in the combination of strains, dosage, trial period, and patient phenotypes (IBS-D, IBS-C, IBS-M) (42). However, variability in study design and strain specificity limits the generalizability of these findings. Additional study using standardized endpoints and mechanistic biomarkers is required in the future to determine the efficacy of therapy. However, variability in study design and strain specificity limits the generalizability of these findings.

#### 2.4.3 Metabolic disorders

Probiotic interventions have been implicated in the suppression of inflammatory biomarkers, lipid profile improvements and a small glycemic control effect on metabolic syndrome or type 2 diabetic patients.

New evidence suggests that probiotics could have an impact on host metabolic homeostasis by controlling bile acid metabolism, short-chain fatty acid synthesis, and metabolic endotoxemia reduction (2). Probiotics can reduce the downgrade inflammation which is one of the primary causes of insulin resistance by improving intestinal barrier integrity and lipopolysaccharide translocation.

Clinical trials have found small yet significant improvements in fasting plasma glucose, HbA1c, total cholesterol, and triglycerides with a regular intake of probiotic-enriched dairy or synbiotic preparations (34, 43). Moreover, the control of the gut-liver axis by probiotics was linked to the decrease of non-alcoholic fatty liver disease biomarkers such as the level of alanine aminotransferase and fat buildup in the liver.

Even though the effect sizes are moderate, addition of probiotics into daily food habits could have cumulative cardiometabolic advantages at the population scale. Nevertheless, the causality needs to be determined and optimal strain-specific interventions have to be defined with large-scale and long-term randomized trials.

#### 2.4.4 Allergic and immune conditions

Probiotic exposure in early life can prevent atopic dermatitis or alter immune development, but long-term effects differ by strain and age of exposure (44, 45). Taken together, these results endorse the use of probiotic-enriched functional foods as preventive health measures in case the selection of strains and dosage is evidence-based.

Recent large-scale meta-analyses point to the fact that the effectiveness of probiotics is significantly different in clinical settings, and there is a strong need to be specific to a particular strain and have a standardized dosing schedule (39, 46). The latest umbrella reviews indicate moderate-certainty evidence in favor of probiotics in the prevention of antibiotic-associated diarrhoea and necrotizing enterocolitis in preterm babies, but the evidence on metabolic syndrome and depression is promising yet heterogeneous (40). The findings highlight the significance of a harmonized trial design and validated biomarkers in subsequent probiotic studies.

### 2.5 Strain specificity and dose–response relationships

The efficacy of probiotics is fundamentally dependent on strain identity and administered dose. Even closely related strains within the same species may exhibit markedly different functional properties due to genomic and metabolic variability. For example, *Lactobacillus rhamnosus* GG differs from other *L. rhamnosus* strains in its adhesion capacity and immunomodulatory effects (47).

Dose–response relationships are equally critical, as sub-therapeutic doses may fail to confer benefits, while excessively high doses may not yield additional advantages. Clinical studies suggest that effective doses generally range between 10^8^ and 10^11^ CFU/day, though optimal dosing varies by strain and indication (11). Furthermore, dosing frequency and duration influence colonization dynamics and sustained efficacy. Therefore, future probiotic-fortified foods must incorporate strain-specific validation and clearly defined dosing regimens to ensure reproducible health outcomes.

## 3 Nutrient delivery systems in probiotic-fortified foods

The effectiveness of probiotic-enriched products critically depends on the construction of delivery systems that maintain the viability of microbes, allow them to survive and be biologically relevant in their interactions with host tissues. The strains of probiotics are also sensitive to environmental factors such as oxygen, thermal, desiccation, gastric acid, and bile salts (48, 49). Hence, efficient delivery plans are needed to ensure the viable counts of ≥10^6^–10^9^ CFU per serving remain intact during the storage and digestion (5).

The delivery systems should achieve three main goals, namely; protection of food during processing and storage, resistance to gastric and bile stress, and control release on intestinal target sites (50, 51). Contemporary approaches to the delivery of probiotics involve encapsulation technologies, food matrix-based vehicles and new responsive systems that are intended to be released at the target point.

In addition to delivery system design, strain identity, viability (expressed as CFU at end of shelf life), and dosing schedule critically determine clinical outcomes. Regulatory and scientific consensus recommend that probiotic products should clearly declare strain designation and viable counts at the end of shelf life rather than at production (52). Furthermore, dosing frequency and duration of consumption influence microbial persistence and functional outcomes, particularly in chronic conditions (53). Therefore, delivery systems must be optimized not only for survival but also for maintaining clinically effective doses throughout product storage and gastrointestinal transit.

### 3.1 Delivery systems

The methods of delivering probiotics can be widely divided into encapsulation methods and integration into food products by using matrices. The choice of a suitable system is based on the characteristics of strains, the physicochemical compatibility with the food system, desired shelf-life, and target population (37, 54).

Oxygen sensitivity is one of the major problems, especially with obligate anaerobic strains like *Bifidobacterium* spp., exposure to moisture and high temperature leads to further decline in viability in storage (55). Therefore, protective mechanisms should reduce oxidative stress, preserve membrane integrity, and sustain metabolic functions.

Notably, probiotic efficacy is identified not just by cell survival but retention of strain-specific functional attributes such as adhesion capacity, SCFA production and immunomodulatory potential (22).

### 3.2 Encapsulation techniques for protection against gastric stress

Encapsulation is one of the most studied methods of enhancing stability of probiotics. This method consists of entrapment of viable cells in protective matrices made of biopolymers or lipid-based materials of food grade that protect against acidity of the stomach and exposure to bile (51, 56).

Some common materials used in microencapsulation include alginate, chitosan, carrageenan, starch derivatives, whey proteins, or gelatin. The use of alginate is especially preferred because of the mild conditions of gelation and biocompatibility. The process of coating the alginate beads with chitosan results in the creation of polyelectrolyte complexes that decrease the porosity and enhance acid resistance (57). Research shows that there is a substantial survival of encapsulated probiotics in simulated gastric conditions relative to the free cells (58).

Liposomes, nanoemulsions and polymeric nanoparticles are some of the nanoscale carriers that are used in nanoencapsulation. These systems increase the interaction of the surface area, enhance the mucosal adhesion, and provide the controlled release mechanisms (59). Nanocarriers made of lipids resist bile salts and allow intestinal delivery to occur in a sustained fashion. PH-sensitive polymeric systems are selectively dissolved by intestinal pH, allowing targeted release (51). Recent developments in the layer-by-layer (LbL) assembly methods enable sequential deposition of oppositely charged biopolymers to increase encapsulation stability and decrease oxygen permeability (60, 61). Moreover, the electrospinning technologies have facilitated the preparation of nanofiber-based probiotic delivery systems that offer greater shield against oxidative stress and gastrointestinal breakdown and a greater surface area to effect controlled intestinal delivery (62). These new generation encapsulation systems demonstrated better survival rates of over 80 percent in simulated conditions of the gastrointestinal conditions, compared to much lower survival of non-encapsulated cells.

The performance of encapsulation is based on the composition of polymers, the crosslinking density, the size of capsules, drying process, and storage (57). Optimized encapsulation enhances a considerable survival of probiotics in simulated gastrointestinal models. The most protective coatings to the gastric tract are nanoencapsulation and pH-responsive, as summarized in Table 1.

**Table 1:** Comparative analysis of probiotic delivery systems with performance metrics.

Delivery system	Protection against gastric acid	Protection against bile salts	Shelf-life stability	Targeted intestinal release	Survival rate (% CFU retention)	Scalability	Advantages	Limitations	References
Free (non-encapsulated) Cells	Low—rapid viability loss at pH<3	Low—strain dependent	Low to moderate	No	<10–20%	High	Simple incorporation; low cost	Poor survival in GI tract	(5, 48)
Microencapsulation (alginate-based)	Moderate to high	Moderate	High	Partial (diffusion-controlled)	40–70%	High	Biocompatible; scalable	Capsule porosity; possible texture impact	(50, 51)
Chitosan-coated alginate capsules	High	High	High	Yes	60–80%	Moderate	Reduced permeability; enhanced acid resistance	Additional processing	(57, 58)
Nanoencapsulation (Liposomes, Nanoemulsions, Nanoparticles)	Very high	High	High	Yes (Controlled release)	>80%	Moderate	Improved mucosal adhesion; sustained delivery	High cost; regulatory complexity	(51, 59)
pH-responsive polymeric systems	Very high	High	High	Yes (Intestinal pH-triggered)	70–85%	Moderate	Site-specific release	Critical polymer selection	(51)
Layer-by-layer (LbL) Systems	High	High	High	Yes (Controlled release)	70–85%	Low–Moderate	Reduced oxygen permeability	Technically complex	(61)
Synbiotic systems (Probiotic + Prebiotic)	Moderate	Moderate	High	Indirect via colonization enhancement	50–70%	High	Synergistic growth stimulation	Compatibility required	(5, 22)
Dairy products (Yogurt, Kefir, Cheese)	Moderate to high (buffering)	Moderate	Moderate to high (refrigerated)	No specific targeting	50–80%	High	Natural buffering; consumer acceptance	Cold chain needed	(54, 55)
Plant-based products (Soy, Oat, Juice)	Low to moderate	Low to moderate	Moderate	No	30–60%	High	Vegan-friendly; market growth	Oxygen sensitivity; low buffering	(37)

### 3.3 Matrix-based delivery systems

Food matrices are natural delivery systems which have effects on probiotic survival and release mechanisms. The dairy-based products like yogurt, kefir, and cheese contain natural buffering power in the form of protein and fat, which shields the cells against acidity in the stomach (54, 55). Cheese, especially, provides a low oxygen exposure and high fat content, which increases long-term viability.

Plant-based alternatives to dairy products such as soy drinks, oat drinks, fruit juices, cereals, and snack items have become popular due to the trends of plant-based diets (37). Nonetheless, such systems are usually characterized by reduced buffering strength and high oxygen permeability, necessitating protective encapsulation mechanisms.

Synbiotic preparations are probiotics that are combined with prebiotic fibers like inulin or fructooligosaccharides. Prebiotics selectively enhance the growth and metabolic activity of probiotics and enhance the chances of survival and colonization (5, 22). Such systems show a higher performance in contrast to single component formulations (Table 1).

### 3.4 Recent advancements in delivery technologies

New technologies are aimed at specific delivery and high stability. pH-sensitive layers based on enteric polymers are used to preserve probiotics in acidic gastric conditions and dissolve in intestinal pH to release to the site (51).

Enzyme-responsive systems make use of surfaces that are destroyed by microbial enzymes in the colon, which allows targeted delivery (59). These technologies are especially applicable to the strains that are aimed at controlling distal colonic microbiota.

High-tech processing (freeze-drying (lyophilization), vacuum drying, and protective cryoprotectants) can be used to improve stability over time, preserving viability (55). High-pressure processing is also explored as the means of enhancing stability without losing functional properties to a significant extent.

Bioengineering approaches have also facilitated the creation of strains that are better in acid tolerance, bile resistance, and adhesion capability, as well as the efficiency of delivery and therapeutic potential (21).

Cold plasma technology has recently appeared as a non-thermal surface decontamination technique that can be used with probiotic fortification. Cold plasma can be used to inactivate the spoilage organisms when optimized, without causing a marked decline in probiotic viability, thereby increasing shelf life (40, 63). Also, microfluidic encapsulation systems can be used to control the distribution of capsule sizes and polymer composition, which increases the reproducibility of the encapsulation system in the context of large-scale production (64). These processing innovations are likely to lower cost barriers and enhance scalability.

### 3.5 Effectiveness of delivery systems

The probiotic delivery systems are dependent on a number of interacting factors to work. Gastric pH is one of the key factors of survival because unprotected cells can barely survive at pH less than 3, and encapsulated cells demonstrate significantly high survival (48). The time of gastric transit also influences the amount of time exposed to acidic conditions. Fatty food matrices have the potential to slow down stomach emptying and provide greater buffering (54). The encapsulation and interaction of the matrices enhance bile salt tolerance by strain (57). Viability is a factor that is highly dependent on storage conditions including temperature, humidity and oxygen exposure. It is more stable when anaerobic strains are kept in packaging that is not permeable to oxygen (55). The interaction between probiotic cells and the constituents of the matrix, such as proteins and polysaccharides, may be enhanced, and it may be beneficial regarding structural protection and functional performance (37). Much testing on simulated gastrointestinal models, shelf-life testing is therefore required in the validation of delivery efficacy. Table 1 shows a comparative data of performance of various systems in terms of protective capacity, where the nanoencapsulation systems and pH-responsive systems have clearly shown better performance.

Furthermore, from a practical food technology perspective, key performance indicators include viable cell counts at the end of shelf life (typically ≥10^6^–10^9^ CFU/serving), percentage survival during storage, and retention of sensory attributes.

Furthermore, practically, as a food technology measure, viable cell counts at the expiry of shelf life (usually ≥10^6^–10^9^ CFU/serving), percentage survival in storage, and retention of sensory properties are considered key performance indicators. For instance, encapsulated probiotics in dairy matrices often retain >10^7^ CFU/g after 4–6 weeks of refrigerated storage (52, 65), whereas non-encapsulated cells may decline below therapeutic thresholds (66). Additionally, processing steps such as pasteurization, drying, and storage temperature significantly affect viability. Sensory attributes such as texture, mouthfeel, and flavor must also be preserved, as larger microcapsules may introduce grittiness, while nanoencapsulation minimizes sensory disruption. These quantitative metrics are critical for translating laboratory findings into commercially viable functional foods.

### 3.6 Consumer acceptance

The economic success of probiotic-enriched functional food will be determined by the sensory quality, perceived value, and perceived transparency of labelling. Encapsulation should not have a negative impact on texture, mouth feel or appearance. Grittiness may be experienced with big microcapsules, but nanoencapsulation does not normally cause sensory alterations (57).

Consumer perception of probiotic-enriched meals is mostly influenced by awareness of functional ingredients and belief in scientific validation, in addition to universal sensory approval. Research has demonstrated that probiotic products are more likely to be adopted by consumers when the health benefits are clearly and effectively communicated and linked to quantifiable outcomes such as relief of the digestive system or defense against illness (5). However, overstated claims or excessive technicalities can undermine trust, so it’s critical to concentrate on evidence-based, legally acceptable communication.

Texture optimization is one of the most significant formulation problems. Systems for microencapsulation should be able to trade minimal sensory disturbance for efficient protection. Nanoencapsulation is done to make the grains feel smaller, but it should be minded that it is apprehensive on the issue of safety perception since some consumers are afraid of nanotechnology in food. The issue of skepticism can be resolved with the help of a clear communication of the materials used, including the ones based on natural biopolymers such as alginate, chitosan, or pectin (37).

Customers are becoming increasingly demanding about natural stabilisers and clean-label products. Biopolymer encapsulation satisfies this need, however artificial additions might not be approved (37). Evidence-based health claims and proper labelling of the viable cell counts are important in assuring consumer confidence and regulatory compliance (5).

The rising significance of short ingredient lists, recognisable substances, and limited processing is what defines the clean label movement (37, 67). Consequently, the producers are reengineering the probiotic delivery systems using plant-based hydrocolloids, resistant starches, and fermentation-based stabilizers rather than employing synthetic emulsifiers (57). It aligns with the broader trends of consumer demands of naturalness, sustainability and responsibility towards the environment (43). In particular, the dairy substitutes and plant-based probiotic drinks have been gaining traction among the younger demographics that harbor a positive perception of plant-based claims in respect to the health and ethics.

Transparency of labels is not limited to viable cell counts but also covers strain identification, storage, and expiration stability, and even the clinically supported dosage levels. New regulatory directions in some jurisdictions currently suggest disclosure at strain level, instead of at the level of the generic species, because probiotic action is strain-specific (5, 39). This accuracy increases consumer choice and decreases deceptive marketing.

Market trends point at the rise of demand of plant-based probiotic foods and synbiotic preparation that combines microbiome and nutrition advantages. Recent market research suggested that synbiotic preparations containing probiotics and prebiotic fibers (i.e., inulin, fructooligosaccharides, resistant dextrin) are becoming more popular because of their dual effect and supposedly higher effectiveness (5, 68). Synbiotics are usually perceived as more complete microbiome solutions by consumers, especially when they are backed by scientific explanation of prebiotic-probiotic synergy.

Moreover, consumer behavior has been greatly influenced by digital health awareness and social media influence. The desire to acquire customized nutrition platforms and microbiome testing services are creating market pressure on customized probiotic products, compelling manufacturers to consider creating targeted formulations to specific demographics, including women, geriatrics, athletes, or pediatrics (69, 70). This trend of personalization could lead to an increased desire to spend money on high quality foods fortified with probiotics in case of relevant evidence.

Acceptance is also dependent on cultural familiarity with fermented foods. Probiotic fortification is commonly seen to be a logical continuation of prior dietary habits in areas where the traditional intake of yogurt, kefir, kimchi, or fermented cereals are being practiced (12). Conversely, markets that are not well accustomed to fermented products might need support through education campaigns in order to gain more knowledge about the workings of probiotics.

Price sensitivity is a crucial factor of large-scale adoption. Though consumers are positive with respect to their attitudes towards functional foods, purchase behaviors are usually limited by the cost differentials with their regular products (67). Thus, the production technologies that can be scaled and the use of cost-efficient encapsulation measures are the key to the fair access and long-term market development (57).

Lastly, the long-term consumer loyalty will be based on the perceived efficacy. When the benefits of consuming probiotic-enriched foods are not experienced, then repeat purchase behavior will reduce. Therefore, not only is it a scientific requirement to align product formulation with clinically proven strains but it is a commercial requirement as well (5, 39).

## 4 Technological innovations in probiotic fortification

**Figure 2:**
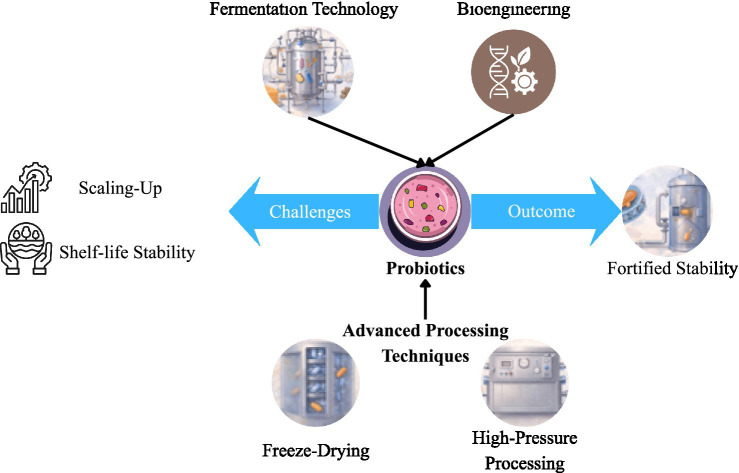
Overview of technological innovations in probiotic fortification, including encapsulation techniques, fermentation strategies, bioengineering approaches, and advanced processing technologies aimed at improving probiotic stability and delivery.

The technological innovation has dramatically changed the approach to probiotic fortification, converting the simple incorporation of microbes to the precision-based stabilization, targeted delivery, and functional improvement. Newer developments in fermentation science, bioengineering and non-thermal processing technologies have enhanced probiotic viability, metabolic activity and scale. Figure 2 summarizes the schemes of these innovations schematically.

### 4.1 Fortification techniques

Probiotic fortification entails addition of live microorganisms into food matrices without affecting stability, safety or sensory characteristics. The conventional methods were mainly based on direct inoculation into fermented dairy products, but the contemporary approaches involve controlled fermentation, encapsulation-mediated incorporation, and post-processing stabilization (Figure 2) (55).

Direct fortification involves attention to the timing of probiotic addition to prevent exposure to deadly thermal treatments. Probiotics are commonly added to dairy systems following pasteurization in cooling stages to maintain viability (54). In the non-dairy system, fortification could be done after processing or through encapsulated formulation to counteract oxygen and pH stress (37).

Fortification with the help of encapsulation also enhances stability, especially with beverages and plant products. It has also been found that co-fortification of micronutrients, bioactive peptides, and prebiotics has been a strategy to increase synergistic functionality (5).

### 4.2 Application of fermentation technology for fortifying dairy and non-dairy foods

**Figure 3:**
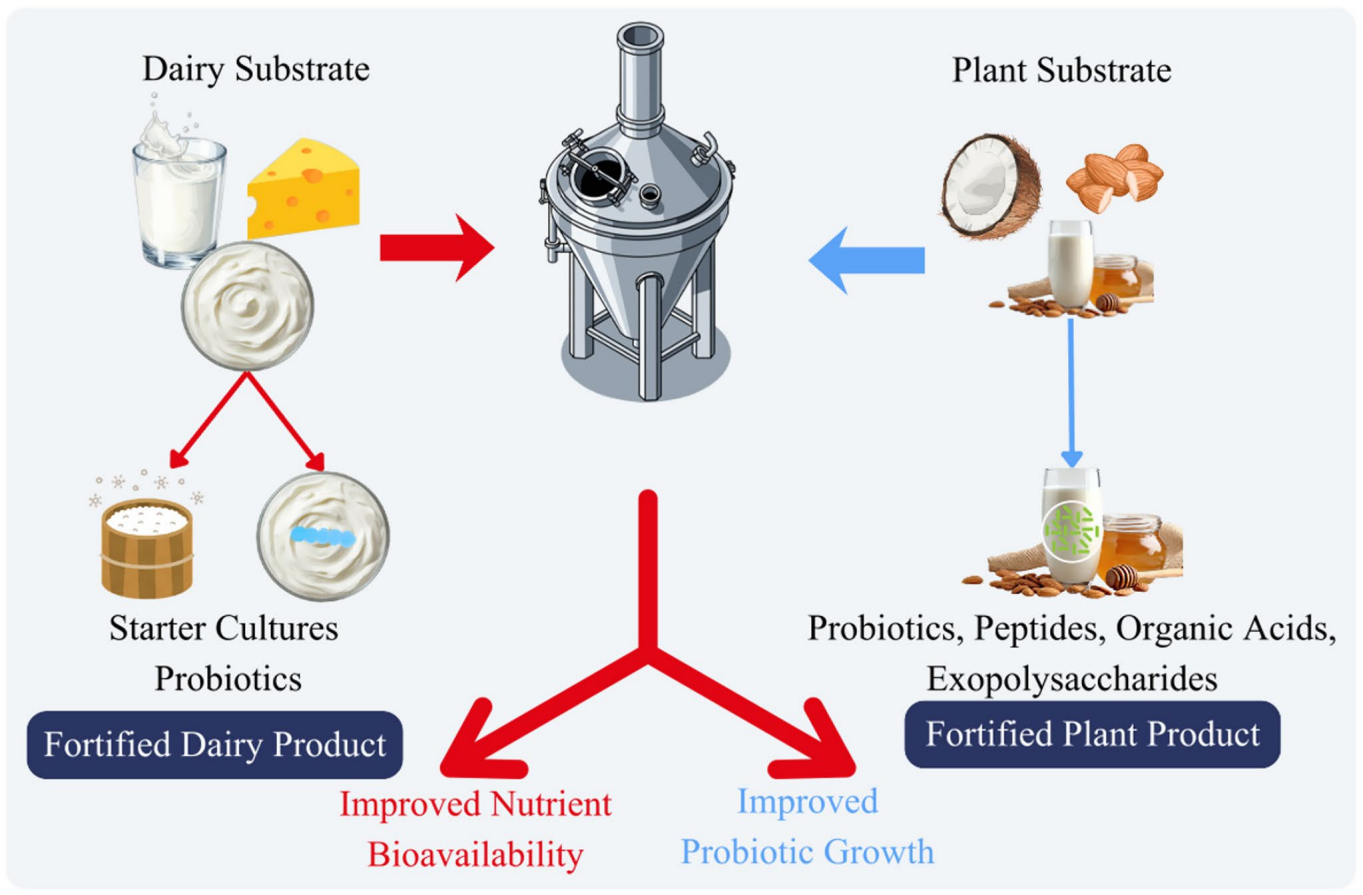
Fermentation-based fortification of dairy and plant-based functional foods, illustrating microbial growth, metabolite production, and enhancement of nutritional and functional properties.

Fermentation has continued to be one of the best technological platforms to fortify probiotics. The mechanism of microbial survival through controlled fermentation is the formation of protective microenvironment abundant in peptides, organic acids, and exopolysaccharides (Figure 3) (12).

Starter cultures are frequently used in dairy products as a combination with probiotic strain to create yogurt, kefir and cheese with increased functional qualities. Exopolysaccharides obtained through fermentation enhance texture, water retention, and microbial stability exopolysaccharides (Figure 3) (54).

Non-dairy fermentation technologies have been extended to plant-based materials like soy, oat, coconut and almond feed exopolysaccharides (Figure 3). Such systems have to be optimized in terms of carbohydrate composition and buffering capacity to maintain the growth of probiotics (37). Fermentation increases bioavailability of nutrients through a reduction in antinutritional factors and an increase in digestibility. Newer fermentation methods combine precision fermentation and controlled fermentation bioreactor methods to maximize biomass production and strain uniformity.

### 4.3 Bioengineering of probiotic strains

The development of molecular biology and synthetic biology has made genetic modification of probiotic strains possible to increase their acid tolerance, resistance to bile, and adhesion ability (21). Probiotics that have undergone genome editing can produce specific therapeutic molecules (like anti-inflammatory peptides), express better mucosal adhesion proteins, increase resistance to oxidative stress, and locally deliver specific metabolites. CRISPR-based genome editing has sped up strain optimization and decreased off-target effects. Engineered strains have demonstrated promise in metabolic control, immune control, and targeted therapeutic uses (71). For example, engineered *Lactococcus lactis* strains expressing interleukin-10 have demonstrated anti-inflammatory effects in preclinical models of colitis, highlighting the therapeutic potential of bioengineered probiotics (71, 72).

Regulatory policies governing genetically modified probiotics, despite their potential success, differ across different countries and are a challenge to commercial translation.

### 4.4 Advanced processing techniques to enhance probiotic stability

Controlled processing and non-thermal technologies have been embraced to improve the stability of probiotics without affecting the viability. Freeze-drying (lyophilization) is still remaining a common technique for long-term preservation (Figure 2). Cryoprotectants (trehalose and skim milk powder) minimize the membrane damage caused by dehydration (73).

High-pressure processing (HPP) is one of the non-thermal preservation techniques that have been explored (Figure 2). Optimized HPP has the ability to inactivate microorganisms that cause spoilage without compromising the survival of probiotics (74).

Spray-drying can be used to provide a cost-effective scaling and can decrease viability unless temperatures are properly managed. Spray-drying post-microencapsulation enhances the survival (Figure 2) (57, 75). These stabilization technologies are as outlined in (Figure 2).

### 4.5 Challenges in probiotic fortification

There are a number of challenges despite the advancement in technology. Stability of shelf-life is a major issue especially when it comes to non-refrigerated products. Exposure to oxygen, migration of moisture and changes in temperature greatly decrease viability (57).

To ensure viability in high scale production, fermentation parameters, drying conditions and packaging systems need to be optimized. Strain specific variation makes standardization difficult. The effects of probiotics are not strain-transferable, and thus strain-specific validation is necessary (5).

Differences in regulations in various countries make commercialization difficult particularly in genetically modified strains. Lastly, the encapsulation and nano-delivery technology is still costly to scale up and this puts a constraint on the wide industrial use of this technology.

## 5 Public health implications of probiotic-fortified foods

Functional foods with probiotics have become a potential instrument of preventive nutrition and population health to decrease the worldwide morbidity of non-communicable and gastrointestinal diseases. There is growing evidence of metabolic, immune, and inflammatory benefits with gut microbiota modulation, which makes probiotics an accessible dietary intervention that has a population-level effect (2, 5).

The clinical efficacy, regulatory harmonization, and accessibility to probiotic-fortified foods by consumers are the determinants of public health integration of probiotic-fortified foods. Regulatory environment Probiotics are regulated differently across different countries and are summarized in Table 2.

**Table 2:** Global regulatory standards for probiotics in functional foods.

Region/country	Regulatory authority	Classification of probiotics	Health claim approval	Minimum viable count requirement	Labelling requirements	Example product	Jurisdiction	Regulatory status	References
United States	FDA (Food and Drug Administration)	Dietary Supplements or Conventional Foods	Structure/function claims allowed; no pre-approval required (unless disease claim)	No fixed national minimum; must meet label claim through shelf-life	Must list strain designation; CFU count at end of shelf-life recommended	Culturelle^®^ (*L. rhamnosus* GG)	USA	Marketed as dietary supplement	(5)
European Union	EFSA (European Food Safety Authority)	Novel Foods or Food Supplements	Strict scientific substantiation required; most probiotic health claims rejected	No universal CFU minimum; must demonstrate safety (QPS status)	Health claims require EFSA authorization; strain specificity required	Actimel® (Danone)	EU	Limited approved claims	(86, 87)
Canada	Health Canada	Natural and Non-prescription Health Products Directorate (NNHPD)	Pre-market approval required for health claims	Typically, 10^9^CFU/day recommended depending on claim	Strain identification and quantity per dose mandatory	Bio-K + ®	Canada	Approved NHP product	(88)
Japan	Consumer Affairs Agency (CAA)	FOSHU (Foods for Specified Health Uses) or FFC	Pre-approved functional claims (FOSHU); notification-based system (FFC)	Product-specific approval; viability must be demonstrated	Clinical evidence required for FOSHU	Yakult®	Japan	FOSHU approved	(89, 90)
China	National Medical Products Administration (NMPA)	Health Foods	Pre-market registration required	Strain-specific evaluation required	Functional claims must be government-approved	Wei Chuan Probiotics Drink	China	Registered health food	(91, 92)
India	FSSAI (Food Safety and Standards Authority of India)	Functional Foods or Nutraceuticals	Permitted strains listed in guidelines	Minimum 10^8^ CFU/g at end of shelf-life recommended	Strain and viable count declaration mandatory	Yakult India®	India	Approved probiotic product	(93, 94)
Australia/New Zealand	FSANZ (Food Standards Australia New Zealand)	Foods or Complementary Medicines	High-level claims require scientific substantiation	No specific CFU mandated; safety required	Must comply with Food Standards Code	Inner Health Plus®	AUS/NZ	Listed complementary medicine	(95–97)

### 5.1 Population-level gut health and disease prevention

Obesity, diabetes type 2, inflammatory bowel disease, the risk of colorectal cancer, and allergic disorders have been linked to dysbiosis (70). Foods fortified with probiotics can provide community-level dietary interventions that can be used to tune gut microbial balance. For instance, clinical trials show a positive effect on the treatment of antibiotic-associated diarrhoea, as well as on the symptoms of irritable bowel syndrome, which leads to a decrease in the burden on healthcare (76). Perspective probiotic exposure in early life might also have an effect on immune maturation and risk of atopic disease (32). Since probiotics may be included into a daily diet, their distribution through food may be more consistent from a public health perspective than supplement-based therapies.

### 5.2 Metabolic health and chronic disease burden

Diabetes type 2 and metabolic syndrome are significant health concerns around the world. An increasing amount of data points to the possibility that taking probiotics may improve lipid metabolism, insulin sensitivity, and systemic inflammation (33, 34).

Probiotics have a mechanistic effect on bile acid metabolism, endotoxemia, and short-chain fatty acid signalling pathways affecting glucose homeostasis (2). Although the effects are usually small, long-term benefits may be accrued through integration into daily diets by fortifying foods.

Longitudinal cohort studies progressively associate habitual consumption of fermented foods with a lower level of systemic inflammatory markers, such as C-reactive protein and interleukin-6 (64, 77). Moreover, mechanistic trials suggest that gut microbial metabolism can be altered to reduce the generation of trimethylamine-N-oxide (TMAO), an emerging cardiovascular risk factor, by the usage of probiotics-enriched foods (43, 78). Despite the fact that causal relationships are yet to be validated, these results indicate cardiometabolic benefits beyond glycemic control.

### 5.3 Immunomodulation and infection control

Foods containing probiotics can improve mucosal immunity and alter the inflammatory response. Some of the strains have been reported to enhance IgA synthesis and cytokine homeostasis (32).

Modulation of gut immunity has been explored as a supporting nutritional measure during outbreaks of infectious diseases, but strong-scale clinical trials have not been conducted extensively. Notably, the issue of safety is paramount in immunocompromised groups.

### 5.4 Accessibility, equity, and nutritional policy

The addition of probiotics to commonly eaten staple foods (e.g., dairy, fermented cereals, plant beverages) creates a prospect of fair public health distribution. Nonetheless, costs and cold-chain provisions can reduce access to low-resource settings (79).

Probiotic formulations and non-dairy vehicles can be shelf-stable, and this could help increase distribution across the globe. The policy frameworks should be able to balance between innovation, safety, correct labelling and prevention of misleading health claims. The biggest obstacle to international standardization can be seen in regulatory heterogeneity, as shown in Table 2.

The international regulatory systems vary widely in the classification of probiotics, including dietary supplements to new foods or therapeutic products. The European Food Safety Authority (EFSA) uses a strict set of standards of health claims, and other regions allow more liberal structure–function claims (5, 80). Unified international definitions of probiotics and minimum viable count would be able to trade globally and enhance consumer transparency. Also, a combination of probiotic fortification of school diets and maternal-child health interventions could be a scalable prevention health measure, especially in areas where gastrointestinal disease is prevalent (81).

### 5.5 Safety considerations in probiotic-fortified foods

Despite their generally recognized safety profile, probiotics are not devoid of risk, particularly in vulnerable populations (5). Key safety concerns include the potential transfer of antibiotic resistance genes, risk of opportunistic infections in immunocompromised individuals, and contamination during large-scale manufacturing processes (82).

Horizontal gene transfer between probiotic strains and pathogenic bacteria may contribute to the spread of antimicrobial resistance, particularly when strains harbor mobile genetic elements (83). Additionally, rare cases of bacteremia and fungemia have been reported in immunocompromised patients receiving probiotic supplementation (84).

Manufacturing-related risks include contamination, misidentification of strains, and variability in viable cell counts. Therefore, stringent quality control, genomic characterization, and adherence to good manufacturing practices (GMP) are essential. Post-market surveillance and adverse event reporting systems should also be strengthened to ensure long-term safety monitoring.

Regulatory frameworks increasingly emphasize strain-level identification, safety validation, and documentation of absence of virulence factors, highlighting the importance of integrating safety assessment into probiotic product development (85).

## 6 Limitations of current evidence and research gaps

Although significant advances have been made in the field of probiotic-enriched functional foods, there are still a number of limitations within the existing body of knowledge. Heterogeneity of the study designs is among the key issues, and it involves the differences in the strain of probiotics, dosing, length of treatment, and the subject of the study, which will make it difficult to compare different studies (5, 12).

Much of the mechanistic information is obtained in *in vitro* and animal work, but with few studies being supported by large human clinical trials (12, 21). This leads to a lack of sufficient clinical support of numerous of the suggested mechanisms, including pathogen exclusion and the regulation of the gut-brain axis (1, 35).

Furthermore, probiotic effects are extremely strain-dependent but numerous studies give results on the species or genus level and can be subject to overgeneralization and misinterpretation of the results. This loss of strain-level specificity is a serious deficiency in applying discovered results into the practical use of functional foods (5, 39).

The lack of consistency in the reported clinical outcomes is another limitation, especially in the case of irritable bowel syndrome, as well as on metabolic conditions, variations in the findings are observed based on the study design and the population characteristics (33, 34). Moreover, there are no standardized biomarkers to evaluate the effectiveness of probiotics, which complicates the comparison of findings between studies (12).

There is also a lack of long-term safety and efficacy data, particularly of next-generation and bioengineered probiotic strains (71, 72). The differences in regulations between regions also make it harder to translate scientific discoveries into commercially acceptable products (5).

Future research should prioritize well-designed randomized controlled trials, standardized reporting frameworks, strain-specific validation, and multi-omics approaches to establish causal relationships and improve clinical applicability (12, 69).

## 7 Conclusion

Probiotic-enriched functional foods are a fast-developing intersection point between microbiome science, food technology, and population health nutrition. The growing mechanistic evidence indicates that probiotics alter the ecology of the gut microbiome, strengthen the integrity of the epithelial barrier, regulate immune responses, and change systemic metabolic and neuroendocrine pathways.

Technological developments such encapsulations, fermentation optimisation, bioengineered strains, and sophisticated stabilisation techniques have significantly improved the viability and delivery effectiveness of probiotics. Nevertheless, the efficacy must be consistent and to achieve this, it is important to select the strain carefully, match it with the matrix, and optimize storage.

In terms of the public health, probiotic-enriched foods provide scalable approaches to the regulation of gut health and have the potential to decrease the burden of metabolic, inflammatory and gastrointestinal diseases. However, the harmonization of regulations, sound clinical validation, and fair accessibility is also one of the most important factors.

The next stage of innovation will probably be shaped by the future integration of multi-omics validation frameworks, next-generation probiotic development, and precision nutrition. Probiotic fortification can be the future of preventive nutrition and functional food systems in the world with further interdisciplinary efforts between microbiologists, food technologists, clinicians, and policymakers.
